# Construction of a ferroptosis-related signature based on seven lncRNAs for prognosis and immune landscape in clear cell renal cell carcinoma

**DOI:** 10.1186/s12920-022-01418-2

**Published:** 2022-12-17

**Authors:** Shi-Yao Wei, Bei Feng, Min Bi, Hai-Ying Guo, Shang-Wei Ning, Rui Cui

**Affiliations:** 1 Department of Nephrology, Second Affiliated Hospital of Harbin Medical University, Harbin, People’s Republic of China; 2 Department of Nephrology, Fourth Affiliated Hospital of Harbin Medical University, 37 Yiyuan Street, Nangang District, Harbin, 150001 Heilongjiang Province People’s Republic of China; 3 College of Bioinformatics Science and Technology, Harbin Medical University, 157 Baojian Road, Nangang District, Harbin, 150081 Heilongjiang Province People’s Republic of China

**Keywords:** ccRCC, Ferroptosis, lncRNA, Prognosis, Immune infiltration

## Abstract

**Background:**

Recent studies have demonstrated that long non-coding RNAs (lncRNAs) are involved in regulating tumor cell ferroptosis. However, prognostic signatures based on ferroptosis-related lncRNAs (FRLs) and their relationship to the immune microenvironment have not been comprehensively explored in clear cell renal cell carcinoma (ccRCC).

**Methods:**

In the present study, the expression profiles of ccRCC were acquired from The Cancer Genome Atlas (TCGA) database; 459 patient specimens and 69 adjacent normal tissues were randomly separated into training or validation cohorts at a 7:3 ratio. We identified 7 FRLs that constitute a prognostic signature according to the differential analysis, correlation analysis, univariate regression, and least absolute shrinkage and selection operator (LASSO) Cox analysis. To identify the independence of risk score as a prognostic factor, univariate and multivariate regression analyses were also performed. Furthermore, CIBERSORT was conducted to analyze the immune infiltration of patients in the high-risk and low-risk groups. Subsequently, the differential expression of immune checkpoint and m6A genes was analyzed in the two risk groups.

**Results:**

A 7-FRLs prognostic signature of ccRCC was developed to distinguish patients into high-risk and low-risk groups with significant survival differences. This signature has great prognostic performance, with the area under the curve (AUC) for 1, 3, and 5 years of 0.713, 0.700, 0.726 in the training set and 0.727, 0.667, and 0.736 in the testing set, respectively. Moreover, this signature was significantly associated with immune infiltration. Correlation analysis showed that risk score was positively correlated with regulatory T cells (Tregs), activated CD4 memory T cells, CD8 T cells and follicular helper T cells, whereas it was inversely correlated with monocytes and M2 macrophages. In addition, the expression of fourteen immune checkpoint genes and nine m6A-related genes varied significantly between the two risk groups.

**Conclusion:**

We established a novel FRLs-based prognostic signature for patients with ccRCC, containing seven lncRNAs with precise predictive performance. The FRLs prognostic signature may play a significant role in antitumor immunity and provide a promising idea for individualized targeted therapy for patients with ccRCC.

**Supplementary Information:**

The online version contains supplementary material available at 10.1186/s12920-022-01418-2.

## Introduction

Renal cell carcinoma (RCC), accounting for more than 90% of renal cancers, is a urological malignancy originating from renal tubular epithelial cells. Clear cell renal cell carcinoma (ccRCC) is the most common subtype among over ten histological and molecular subtypes of RCC, making up about 65–75% of RCC [1, 2]. The main treatment for ccRCC is surgery; however, many surgical resection is not possible for many patients at the time of initial diagnosis or develop postoperative metastasis. Therefore, the overall treatment outcome in patients with ccRCC remains unsatisfactory [3]. In the last decade, the overall survival of patients with ccRCC has been significantly ameliorated due to the use of immunosuppressive drugs; however, immunosuppressive therapy cannot benefit all patients due to drug resistance. Therefore, the overall treatment effect remains unsatisfactory [4, 5]. Thus, it is worth investigating the underlying mechanisms and effective prognostic methods for ccRCC, developing promising targeted, personalized therapies.

Ferroptosis, a novel programmed cell death process, is triggered by extra-mitochondrial lipid peroxidation caused by an increase in iron-dependent reactive oxygen species [6]. Ferroptosis is distinguished from programmed cell death due to its unique morphological and biological characteristics [7]. Recent evidence has confirmed that ferroptosis plays crucial roles in the development, invasion, metastasis, and treatment resistance of cancer to varying degrees [8]. Studies have shown that ferroptosis inhibition accelerates glioma proliferation and metastasis, malignant transformation, and angiogenesis [9, 10]. A previous study has shown that ACSL4, a positive activator of ferroptosis, is overexpressed in multiple cancer types. ACSL4 knockdown reduced 17β-estradiol-induced migration, proliferation, and invasive properties of cancer cells, whereas ACSL4 overexpression enhanced tumor growth and proliferation [11]. Fu et al. demonstrated that promoting ferroptosis alleviates cisplatin resistance in gastric cancer cells, presenting a potential approach for improving the outcomes of chemotherapy for gastric cancer [12]. These results support the view that ferroptosis induction may be a prospective anti-cancer therapeutic strategy [13]. However, reports on the role of ferroptosis regulation in the prognosis and treatment of ccRCC are limited. Therefore, it is important to explore the prognostic and therapeutic biomarkers related to ferroptosis in ccRCC. Long non-coding RNAs (lncRNAs), a novel class of non-coding RNAs, are more than 200 nucleotides in length. Increasing evidence has confirmed that a great deal of lncRNAs is involved in important pathophysiological processes in a variety of cancers, such as cell growth, migration, invasion, and apoptosis [14]. LncRNAs have been considered promising therapeutic targets for cancer treatment because of their unique expressive characteristics [15]. Luo et al. confirmed that lncRNA RP11-89 enhances ferroptosis resistance and promotes tumorigenesis in bladder cancer by sponging miR-129-5p [16]. Wang et al. demonstrated that blocking LINC00336 expression attenuates cell growth and aggregation as well as tumorigenesis while inducing ferroptosis [17]. Nevertheless, the regulatory role of lncRNAs with respect to ferroptosis in ccRCC still requires further investigation, and the value of ferroptosis-related lncRNAs (FRLs) as prognostic biomarkers in patients with ccRCC has not been systematically evaluated. Currently, immune checkpoint blockade therapies are the major strategy for ccRCC immune-targeted treatments. It has been suggested that the significant clinical benefits of immunotherapy may stem partly from triggering ferroptosis in tumor cells [18]. Wang et al. confirmed that promoting ferroptosis in tumor cells is an essential requirement for immune checkpoint therapy for blocking PD-L1 [19]. The N6-methyladenosine (m6A) modification plays an important role in biological processes (BPs), including proliferation, migration, as well as infiltration [20]. Therefore, it is essential to elucidate the association between m6A modifications and ferroptosis in ccRCC. Based on transcriptome data from The Cancer Genome Atlas (TCGA), we constructed a prognostic signature based on FRLs by univariate, multifactorial, and least absolute shrinkage and selection operator (LASSO) Cox regression to predict the overall survival and immune invasion status of patients with ccRCC. At the same time, receiver operating characteristic (ROC) curves and decision curve analysis (DCA) were used to evaluate the performance of the prognostic signature. Additionally, we analyzed the relationship between the prognostic risk score and the expression of immune checkpoint and m6A genes in patients with ccRCC. Therefore, our study aimed to provide a more accurate prognostic prediction for ccRCC patients through this signature, providing a potential direction for individualized, targeted therapies.

## Materials and methods

### Datasets sources and processing

The high throughput sequencing data ccRCC from TCGA and the related clinical parameters were acquired from the UCSC Xena genome browser (https://xena.ucsc.edu) [21]. The annotation profile, version for human release 22, and mapping probes to gene symbols were obtained from the GENCODE database (https://www.gencodegenes.org) [22]. Clinical data and corresponding expression data included in the analysis were filtered based on the following criteria: (i) patients with complete clinical information and (ii) patients with an overall survival time of ≥ 60 days. A total of 564 samples (495 ccRCC and 69 adjacent normal tissues) were included in the final analysis. In addition, the expression profile values were normalized to transcripts per million values. Genes with no expression in more than 100 samples were removed. The ccRCC samples were randomly divided into training and testing sets at a ratio of 7:3. Patients’ clinical characteristics are summarized in Table 1. Moreover, a gene list consisting of 259 ferroptosis-related genes was obtained from version 1 of the FerrDb database (http://www.zhounan.org/ferrdb; Additional file 1: Table S1) [23].

**Table 1:** The clinical characteristics of patients in training set and testing set from TCGA dataset

Characteristics	Training cohort (N = 346)	Testing cohort (N = 149)	Entire TCGA cohort (N = 495)	*p*
Age (years)				0.695
< 60	168 (48.6%)	69 (46.3%)	237 (47.9%)	
≥ 60	178 (51.4%)	80 (53.7%)	258 (52.1%)	
Gender				0.256
Female	124 (35.8%)	45 (30.2%)	169 (34.1%)	
Male	222 (64.2%)	104 (69.8%)	326 (65.9%)	
Grade				0.093
G1	8 (2.3%)	2 (1.3%)	10 (2.0%)	
G2	164 (47.4%)	54 (36.2%)	218 (44.0%)	
G3	130 (37.6%)	68 (45.6%)	198 (40.0%)	
G4	44 (12.7%)	25 (16.8%)	69 (13.9%)	
Stage				0.298
Stage i	171 (49.4%)	77 (51.7%)	248 (50.1%)	
Stage ii	39 (11.3%)	14 (9.4%)	53 (10.7%)	
Stage iii	87 (25.1%)	29 (19.5%)	116 (23.4%)	
Stage iv	49 (14.2%)	29 (19.5%)	78 (15.8%)	
T				0.923
T1	175 (50.6%)	79 (53.0%)	254 (51.3%)	
T2	47 (13.6%)	18 (12.1%)	65 (13.1%)	
T3	118 (34.1%)	49 (32.9%)	167 (33.7%)	
T4	6 (1.7%)	3 (2.0%)	9 (1.8%)	
N				0.927
N0	156 (45.1%)	68 (45.6%)	224 (45.3%)	
N1	10 (2.9%)	3 (2.0%)	13 (2.6%)	
NX	180 (52.0%)	78 (52.3%)	258 (52.1%)	
M				0.410
M0	283 (81.8%)	115 (77.2%)	398 (80.4%)	
M1	47 (13.6%)	27 (18.1%)	74 (14.9%)	
MX	16 (4.6%)	7 (4.7%)	23 (4.6%)	

*p* values were calculated using the fisher exact test

### Ferroptosis-related differentially expressed genes and functional enrichment analysis

mRNA and lncRNA data were extracted from the total expression profile of TCGA RNA-sequencing data, and the “limma” package was applied for differential expression analysis [24]. The |log_2_-fold change (FC)|> 1 and *p* value < 0.05, adjusted by the Benjamini–Hochberg method, were defined as the criteria to identify differentially expressed genes (DEGs) or differentially expressed lncRNAs (DE-lncRNAs). DEGs were intersected with 259 ferroptosis-related genes to screen out ferroptosis-related DEGs. Gene Ontology (GO) and Kyoto Encyclopedia of Genes and Genomes (KEGG) analysis are widely used enrichment analysis methods [25–27]. In this study, GO terms were used to annotate biological terms, including biological processes (BPs), cellular components (CCs), and molecular functions (MFs). Moreover, the KEGG was used to map the biological pathways. To annotate the biological function of ferroptosis-related DEGs, GO annotation and KEGG enrichment analyses were performed. Gene symbols were converted to Entrez ID using the “org.Hs.eg.db” package, and then enrichment analysis was conducted depending on the “clusterProfiler” package [28].

### Development of the FRLs-based prognostic signature

In order to evaluate the correlation between ferroptosis-related differentially expressed genes and DE-lncRNAs in ccRCC, the Pearson correlation coefficient was calculated. |R^2^|> 0.4 at *p* < 0.01 was considered significant. A total of 65 lncRNA-gene pairs (Additional file 2: Table S2) were obtained, including 56 lncRNAs, which were defined as FRLs. Univariate Cox regression and LASSO Cox regression analyses were conducted on the 56 lncRNAs. Finally, seven lncRNAs with non-zero regression coefficients were obtained to develop the prognostic signature, which was built using the following formula:

$${\mathrm{Risk\,score}}=\sum_{{\mathrm{i}}=1}^{{\mathrm{n}}}{\mathrm{coef}}\_{\mathrm{i}}\times {\exp}\_{\mathrm{i}}$$

where *n* represents the total number of screened prognostics lncRNAs, while ${\mathrm{coef}}\_{\rm i}$ and $\mathrm{exp}\_{\rm i}$ represent the regression coefficient and expression value of the *i*th ${\mathrm{lncRNA}}$, respectively.

### Survival analysis and model evaluation

Using the median risk score as a threshold, the sample was divided into high-risk (>median number) and low-risk (<median number) groups. Then we separately compared the overall survival of the two groups in the training and validation cohorts and presented them in the form of Kaplan–Meier survival curves (KM curves) through the “survminer” package. Similarly, KM curves were also used to evaluate the prognostic value of seven FRLs constituting the prognostic signature in patients with ccRCC. In addition, univariate and multivariate Cox regression analyses of the clinical variables and risk models were conducted to assess the effectiveness of the risk model. Then, the excellent predictive efficiency of the risk model, compared with other clinical parameters, was demonstrated as time-dependent ROC curves. Finally, DCA, a method widely used to evaluate performance of models, was used to assess the clinical utility of our prognostic models. The DCA results depend on the “survival” and “dcurves” packages presented as decision curves. All of the above analyses were conducted independently in the training and testing sets. The presentation of univariate and multivariate Cox analyses and the ROC curves were realized with “forestplot” and “timeROC” packages.

### Evaluation of infiltrating immune cells in ccRCC

The CIBERSORT algorithm was used to assess the immune cell infiltration rates in the two risk groups. The reference dataset for the CIBERSORT algorithm is a matrix named LM22 consisting of 547 leukocyte gene signatures. Based on LM22, CIBERSORT can robustly characterize 22 immune cells from expression profiles; thus, CIBERSORT is widely used in the analysis of tumor microenvironment (TME) [29]. We analyzed the mRNA expression matrix using the CIBERSORT R script (https://cibersort.stanford.edu/) obtained from the official website of CIBERSORT; the LM22 signature gene file and 1000 permutations were employed as the reference. The difference in immune cell infiltration levels between the high-risk and low-risk groups was calculated by the Wilcoxon signed-rank sum test (*p* < 0.05).

### Analysis of immune checkpoints genes and m6A-related genes

To explore the potential significance of the FRLs signature in immunotherapy, we analyzed the differences in the expression of 14 candidate immune checkpoint genes extracted from literature (*PDCD1* (*PD-1*), *CD274* (*PD-L1*), *CTLA4*, *ADORA2A*, *C10orf54* (*VISTA*), *HAVCR2* (*TIM-3*), *ICOSLG*, *NT5E*, *CD27*, *IDO2*, *LAG3*, *TIGIT*, *TNFRSF18*, and *TNFRSF9*) in tumor immunotherapy between high-risk and low-risk patients [30–40]. Additionally, increasing evidence has confirmed that m6A-related genes play an essential role in tumor proliferation, migration, and invasion; therefore, these genes are expected to be potential targets for tumor therapy. Therefore, we collected ten key m6A genes, including three writers (*METTL3*, *METTL14*, and *WTAP*), two erasers (*FTO* and *ALKBH5*), and five readers (*YTHDF1*, *YTHDF2*, *YTHDF3*, *YTHDC1*, and *YTHDC2*), from the literature and analyzed their expression in the high-risk and low-risk groups [20]. The results are presented in the form of split violin plots depending on the R package “ggplot2”; *p* < 0.05 indicates a significant difference in gene expression between the two groups**.**

### Statistical analysis

Differences between two variables were analyzed using the Wilcoxon signed-rank sum test, and differences between multiple variables were analyzed using the Kruskal–Wallis test. All statistical analyses were performed using the R software (version 4.0.3), and significance was set at *p* < 0.05. If necessary, *p*-values were adjusted using the Benjamini–Hochberg or false discovery rate methods.

## Results

### Ferroptosis-related DEGs and functional enrichment analysis

**Fig. 1:**
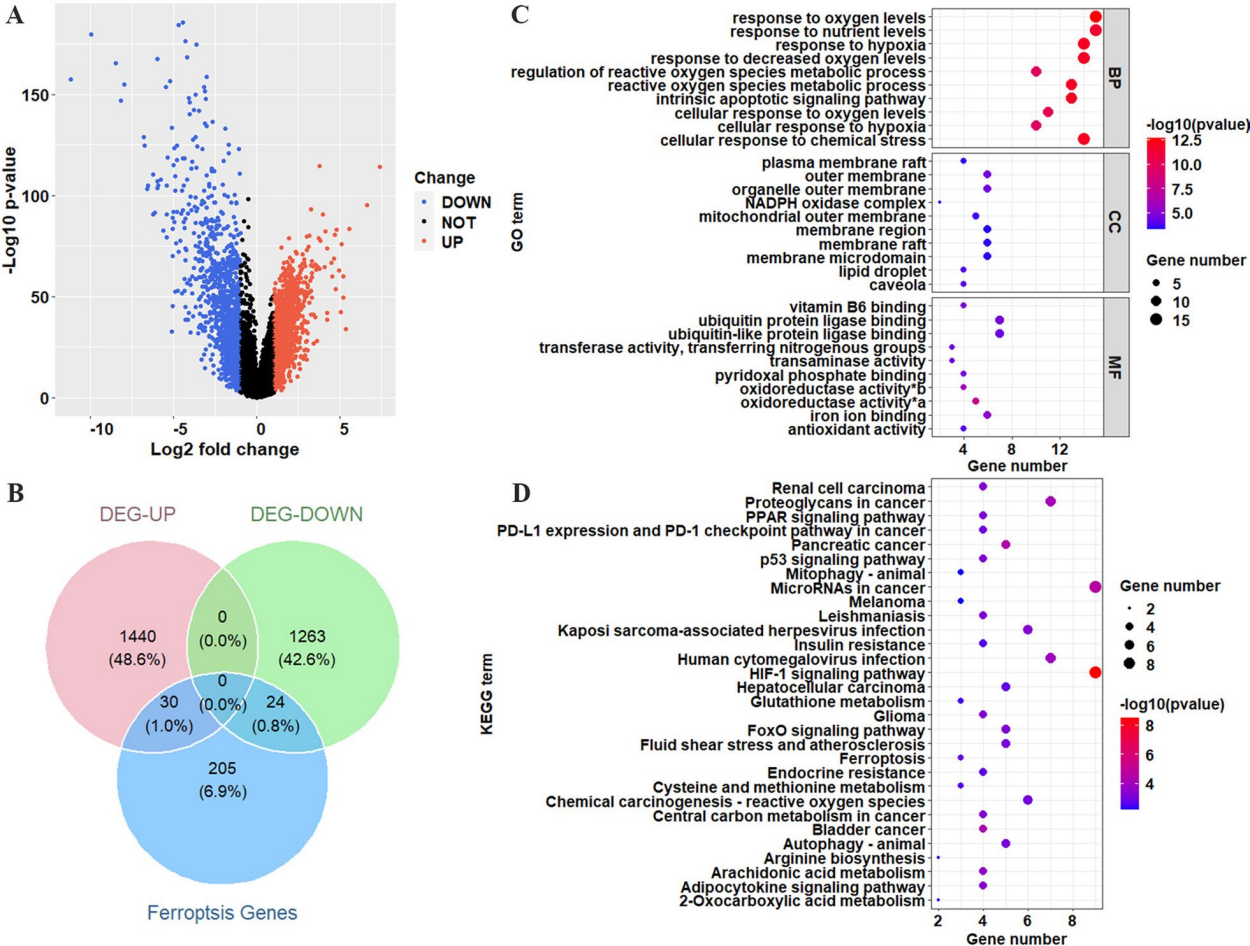
Identification and enrichment analysis of ferroptosis genes for ccRCC. **A** Volcano plots showing the DEGs between ccRCC tissue and normal tissue; the blue dots represent down-regulated genes, the red dots represent up-regulated genes, and the black dots indicate genes with no differential expression. *p* < 0.05, |FC| > 2. **B** Venn diagram of differential genes and ferroptosis genes. **C** Top 10 terms of BP, MF, and CC in GO analysis for differentially expressed ferroptosis genes. **D** Top 30 terms of KEGG analysis for DE-ferroptosis genes. oxidoreductase activity*a: oxidoreductase activity, acting on single donors with incorporation of molecular oxygen, oxidoreductase activity*b: oxidoreductase activity, acting on single donors with incorporation of molecular oxygen, incorporation of two atoms of oxygen

After extracting the mRNA data of the training set for differential expression analysis, 2757 DEGs, consisting of 1470 upregulated and 1287 downregulated genes, were obtained (Fig. 1A). Fifty-four ferroptosis-related DEGs were identified by intersecting DEGs with 259 ferroptosis genes downloaded from FerrDb, including 108 drivers, 69 suppressors, and 111 markers (Fig. 1B). To annotate the function of ferroptosis-related DEGs, GO annotation and KEGG enrichment analysis were carried out. The top 10 GO terms for BP, MF, and CC were visualized in a dot plot (Fig. 1C). Ferroptosis-related DEGs were significantly enriched in response to oxygen levels, nutrient levels, hypoxia, decreased oxygen levels, reactive oxygen species, and metabolic process in BPs, vitamin B6 binding, ubiquitin-protein ligase binding, ubiquitin-like protein ligase binding, transaminase activity, pyridoxal phosphate binding in MFs, plasma membrane raft, outer membrane, outer organelle membrane, NADPH oxidase complex, and mitochondrial outer membrane in CCs. The KEGG pathways results showed that ferroptosis-related DEGs were significantly enriched in RCC, proteoglycans in cancer, PPAR signaling pathway, PD-L1 expression, and PD-1 checkpoint pathway in cancer. The dot plot is shown in Fig. 1D.

### Construction of the prognostic FRLs signature

**Fig. 2:**
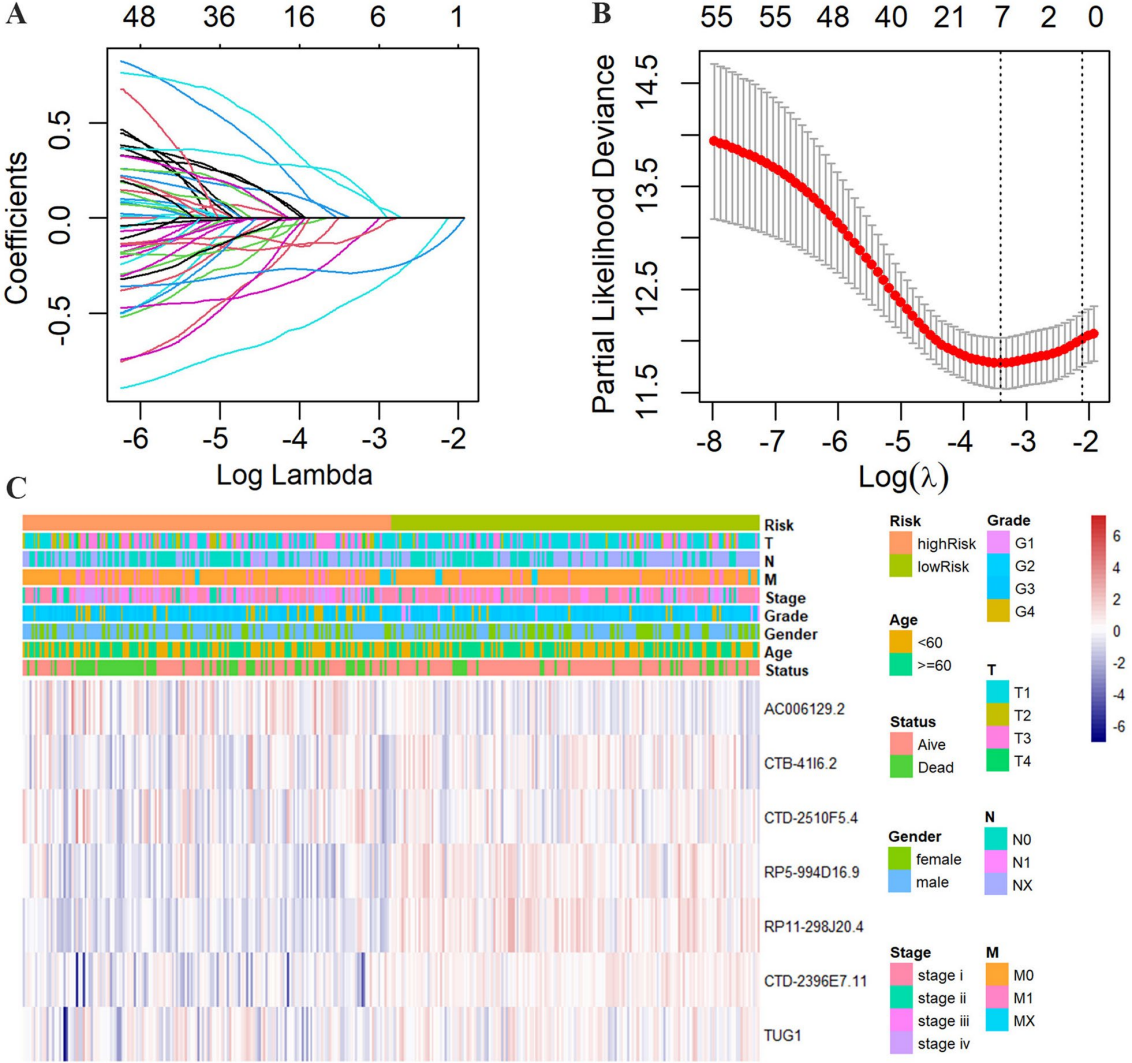
Construction of FRLs prognostic signature. **A** Coefficients of the LASSO regression model. **B** The LASSO regression model obtains seven prognostic lncRNAs with a minimum lambda value. **C** Heatmap of the associations among the expression levels of seven ferroptosis-related lncRNAs, clinical features, and clinicopathological parameters

To uncover FRLs, we extracted lncRNA expression data from the training set and calculated Pearson’s correlation coefficients between ferroptosis-related DEGs and lncRNAs. |R^2^|> 0.4 at *p* < 0.01 was regarded as the significant threshold. Finally, 65 lncRNA-gene pairs were filtered out, including 56 lncRNAs. The 56 lncRNAs were defined as FRLs. To screen lncRNAs with great prognostic value, we performed LASSO Cox regression on 56 FRLs with tenfold cross-validation. Finally, a prognostic signature composed of seven non-zero coefficient lncRNAs (AC006129.2, CTB-41I6.2, CTD-2510F5.4, RP5-994D16.9, RP11-298J20.4, CTD-2396E7.11, and TUG1) was constructed (Fig. 2A–B). The distributions of the seven lncRNAs and clinicopathological factors are shown in Fig. 2C. To evaluate the prognostic value of the seven FRLs in patients with ccRCC, Kaplan–Meier analyses were conducted. The results showed that the patients with ccRCC and high CTD-2396E7.11, RP11-298J20.4, RP5-994D16.9, and CTB-41I6.2 expression had better prognoses, while those with high AC006129.2, CTD-2510F5.4, and TUG1 expression had worse prognoses (Additional file 4: Figure S1; Additional file 5: Figure S2).

### Evaluation of the efficiency of the prognostic signature based on FRLs

**Fig. 3:**
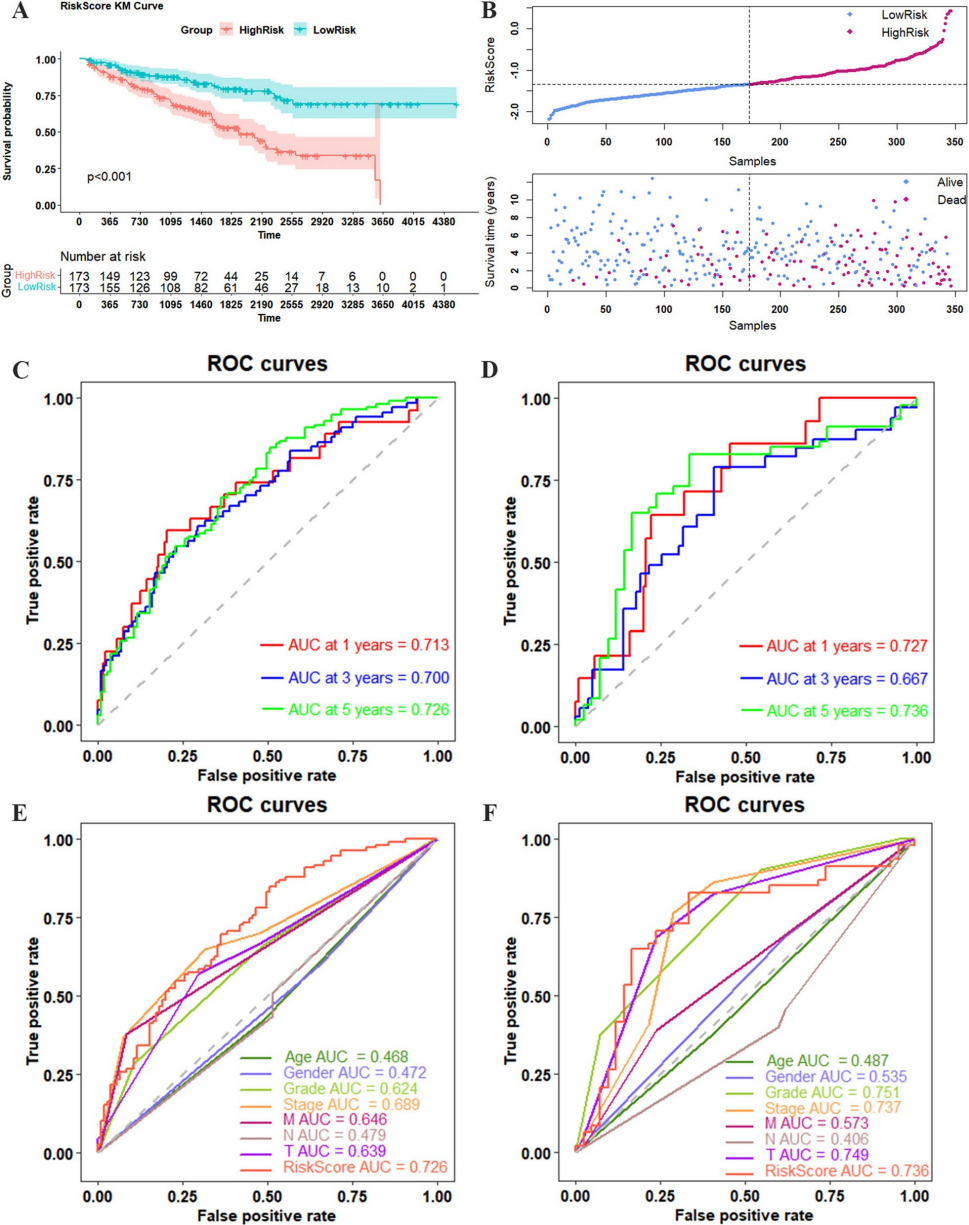
Evaluation of prognostic signature efficiency. **A** KM curves showing a significant difference in survival between the two groups of patients. **B** Survival curve and survival status plot of ccRCC patients. 1, 3, and 5 years prognostic ROC curves of the training set (**C**) and validation set (**D**). ROC curves of risk score and clinical parameters in the training set (**E**) and validation set (**F**)

On the basis of the risk score, patients with ccRCC were stratified into high-risk and low-risk groups, according to the median risk score cutoff. Kaplan–Meier analysis and survival status charts indicated worse prognoses in the high-risk group than in the low-risk group (Fig. 3A–B). In the training and validation sets, we analyzed the sensitivity and specificity of the prognostic model for patient survival at 1, 3, and 5 years, using time-dependent ROC curves. The results indicated that the prognosis model has a great predictive ability for 1, 3, and 5 years survival rates, with respective areas under the curve (AUC) of 0.713, 0.700, and 0.726 in the training cohort, and 0.727, 0.667, and 0.736 in the testing cohort, respectively (Fig. 3C–D). To further assess the efficiency of the risk model, we compared the predictive power of the risk score with multiple clinical parameters, such as age, gender, grade, AJCC stage, T stage, N stage, and M stage. The results showed that the prognostic risk score was more predictive than the clinical variables (Fig. 3E–F). In addition, to evaluate the clinical utility of the prognostic risk model, we conducted a 1, 3, and 5 years DCA analysis of the training and validation sets. The results indicated that our prognostic risk model has better clinical utility than clinical parameters such as age, gender, grade, AJCC stage, T stage, N stage, and M stage (Additional file 6: Figure S3).

### Verification of independent prognostic risk score model

**Fig. 4:**
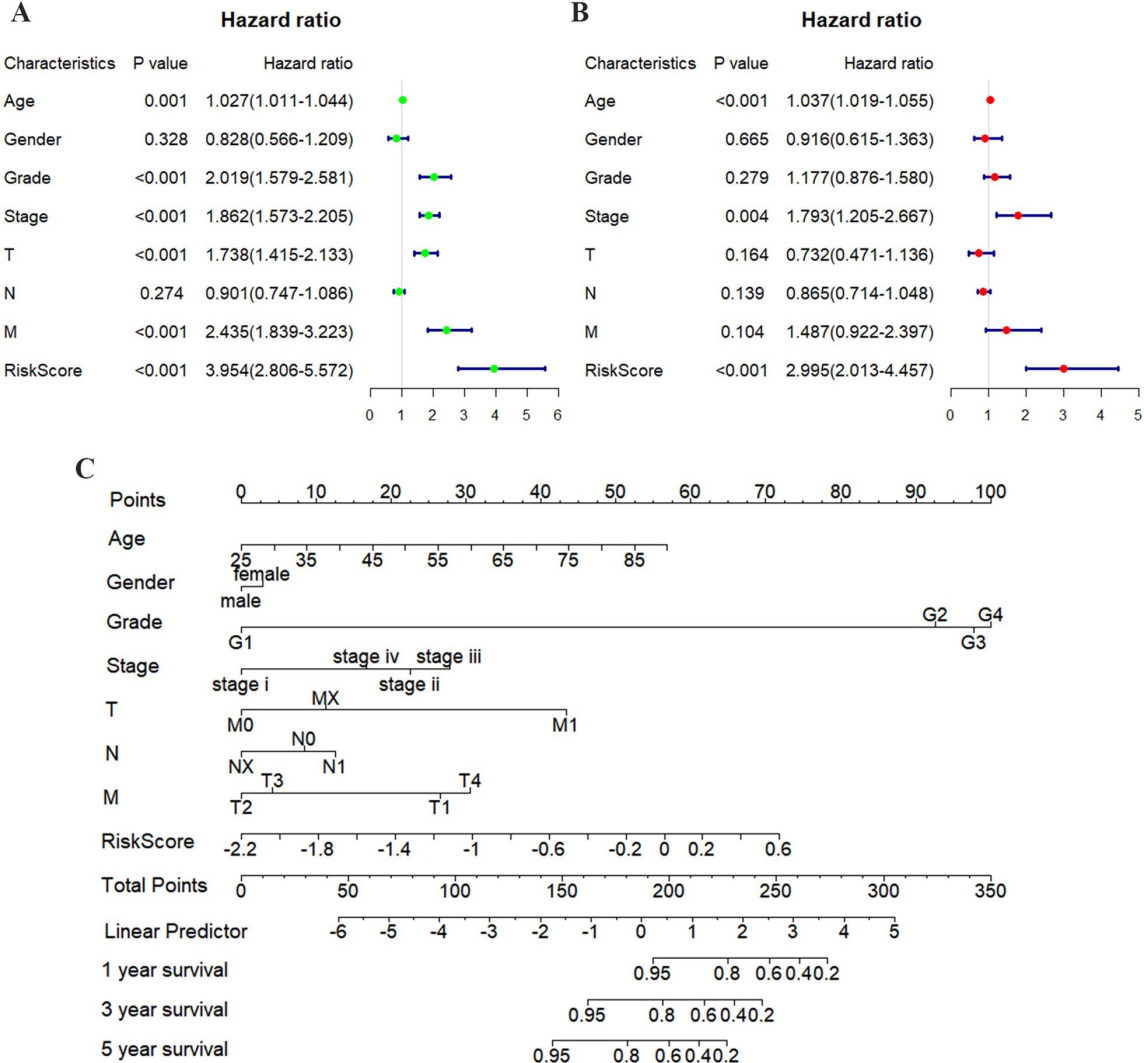
Clinical value of risk score by independent prognostic analysis in ccRCC patients. **A** Univariate Cox regression analysis. **B** Multivariate Cox regression analysis. **C** Nomogram with various clinical parameters and risk scores

Univariate regression analysis was conducted to identify independent prognostic factors, and the results indicated that the risk score (HR: 3.954, 95% CI: 2.806–5.572, *p* < 0.001) was an independent prognostic factor. In addition, age (HR: 1.027, 95% CI: 1.011–1.044, *p* = 0.001), grade (HR: 2.019, 95% CI: 1.579–2.581, *p* < 0.001), AJCC stage (HR: 1.862, 95% CI: 1.573–2.205, *p* < 0.001), T stage (HR: 1.738, 95% CI: 1.415–2.133, *p* < 0.001), and M stage (HR: 2.435,95% CI: 1.839–3.223, *p* < 0.001) were independent prognostic risk factors (Fig. 4A). Multivariate regression analysis was performed, highlighting three independent prognostic risk factors: age (HR: 1.037, 95% CI: 1.019–1.055, *p* < 0.001), AJCC stage (HR: 1.793, 95% CI: 1.205–2.667, *p* = 0.004), and risk score (HR: 2.995, 95% CI: 2.013–4.457, *p* < 0.001; Fig. 4B). Finally, a nomogram, which integrated clinical parameters and risk scores, was constructed to forecast patient survival over periods of 1, 3, and 5 years (Fig. 4C).

### Immune infiltration analysis in ccRCC

**Fig. 5:**
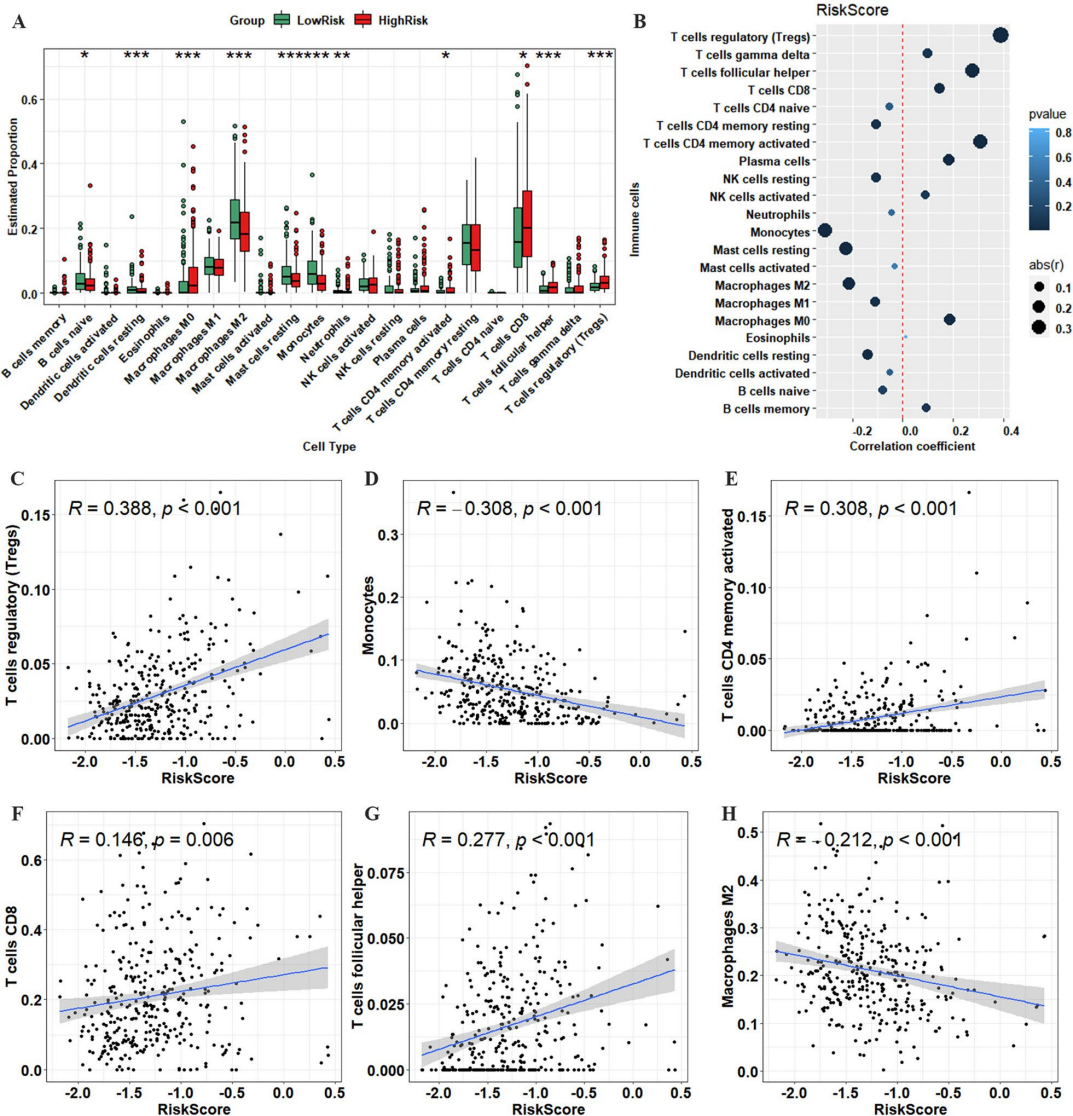
Analysis of immune infiltration in ccRCC patients. **A** Boxplots showing infiltration of immune cells in ccRCC patients from the two groups. **B** Bubble plots representing the correlations between immune cells and the risk score. Scatter plot of the correlation between the risk score and various immune cells, including regulatory T cells (Tregs) (**C**), monocytes (**D**), activated CD4 memory T cells (**E**), CD8 T cells (**F**), follicular helper T cells (**G**), and M2 macrophages (**H**). *p < 0.05, **p < 0.01, ***p < 0.001

To further explore the immune infiltrating component between the two groups, CIBERSORT was used to measure the infiltration proportions of 22 immune cell types in the high-risk and low-risk groups. The low-risk group had a higher proportion of M2 macrophages, naive B cells, dendritic cells, monocytes, resting mast cells, and neutrophils than the high-risk group. In contrast, the proportions of M0 macrophages, regulatory T cells (Tregs), activated CD4 memory T cells, CD8 T cells, and follicular helper T cells were higher in the high-risk group (Fig. 5A). We also performed CIBERSORT analysis on the training set, and the results showed higher invasion proportions of M2 macrophages, mast cells resting, monocytes, and neutrophils in the low-risk group. In comparison, activated CD4 memory T cells, regulatory T cells (Tregs), and follicular helper T cells showed higher infiltration rates in the high-risk group (Additional file 7: Figure S4). We also explored the correlation between the risk score and 22 immune cell types; the results are shown as a dot plot in Fig. 5B. Moreover, the risk score was remarkably correlated with multiple immune cell infiltration in patients with ccRCC, such as Tregs (R = 0.388, *p* < 0.001), monocytes (R = −0.308, *p* < 0.001), activated CD4 memory T cells (R = 0.308, *p* < 0.001), CD8 T cells (R = 0.146, *p* = 0.006), follicular helper T cells (R = 0.277, *p* < 0.001), and M2 macrophages (R = −0.212, *p* < 0.001; Fig. 5C–H).

### Expression of immune checkpoints genes and m6A genes

**Fig. 6:**
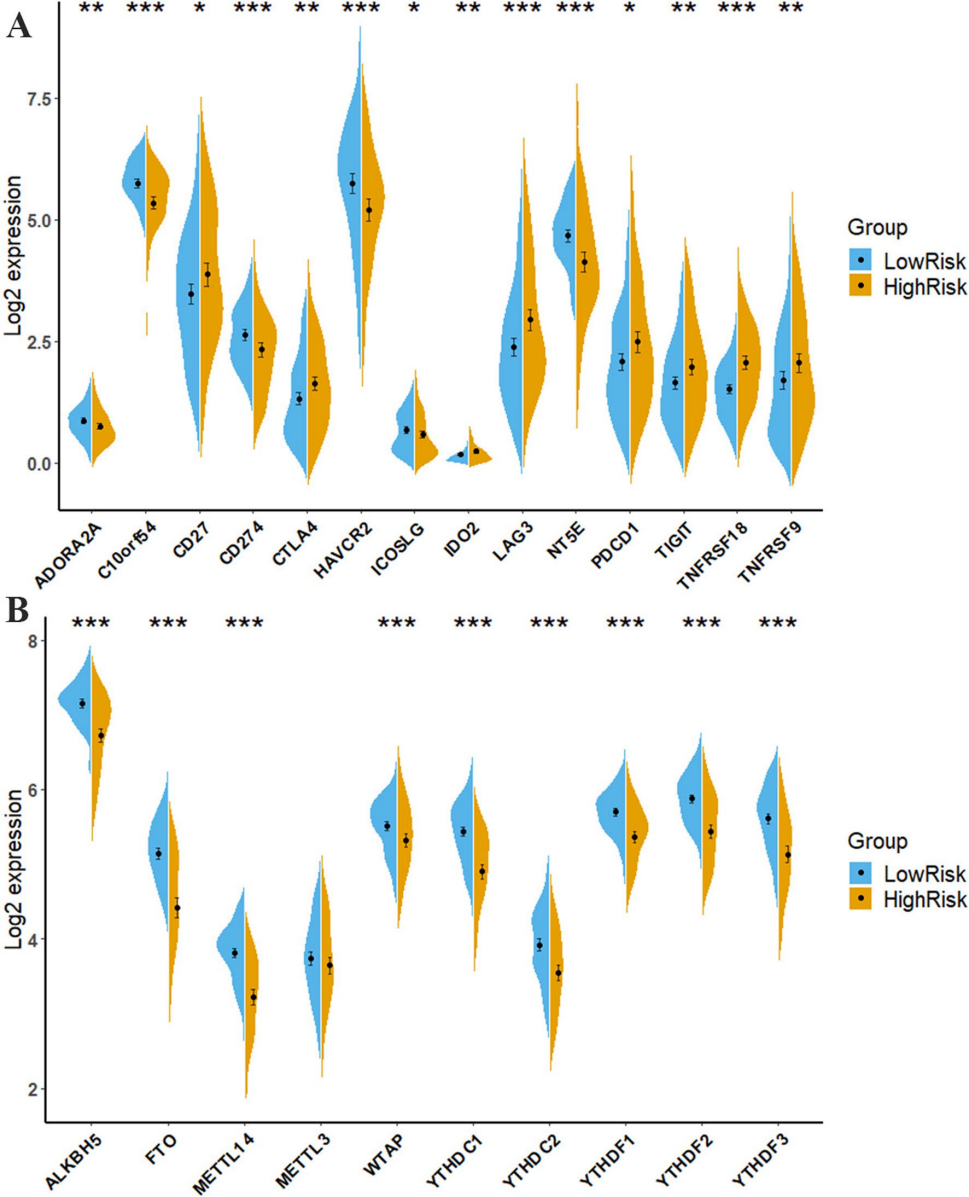
Expression of m6A genes and immune checkpoints. Splitviolin plots indicating the differential expression of ICGs (**A**) and m6A genes (**B**) between high-risk and low-risk patients. *p < 0.05, **p < 0.01, ***p < 0.001

T cells are the main immune effector cells in the tumor microenvironment of ccRCC [41] and are closely associated with the risk score in our analysis; therefore, we further characterized the expression of 14 immune checkpoint genes in the high-risk and low-risk groups of patients with ccRCC. We found that the expression of six immune checkpoint genes was higher in the low-risk group, including *ADORA2A*, *C10orf54*, *CD274* (*PD-L1*), *HAVCR2*, *ICOSLG*, and *NT5E*, whereas the other eight immune checkpoint genes (*CD27*, *CTLA4*, *IDO2*, *LAG3*, *PDCD1* (*PD-1*), *TIGIT*, *TNFRSF18*, and *TNFRSF9*) were highly expressed in the high-risk group (Fig. 6A). These results indicated that the risk score might be a potential assessment tool for immunotherapies in patients with ccRCC. Many studies have focused on m6A, which is the most prevalent form of mRNA modification and is involved in disease occurrence and development [42]. Notably, the difference in the expression levels of ten m6A-related genes (*ALKBH5*, *FTO*, *METTL14*, *WTAP*, *YTHDC1*, *YTHDC2*, *YTHDF1*, *YTHDF2*, and *YTHDF3*) between the two risk groups is presented in Fig. 6B. As presented in Fig. 6B, the expression of nine genes was significantly higher in the low-risk group, except for METTL3 (*p* = 0.309). These results may provide a novel viewpoint for researching m6A-related genes in ccRCC.

## Discussion

ccRCC, the most common type of renal cancer worldwide, has a high mortality rate [1]. Primary tumor resection remains the primary ccRCC treatment; however, approximately 20–50% of the patients still develop metastasis after surgery, leading to a poor overall prognosis of ccRCC [43]. Although immune checkpoint inhibition therapy has progressed in recent decades, most patients experience disease progression due to varying degrees and types of drug resistance [1, 44]. Therefore, it is urgent to develop powerful prognostic prediction tools and personalized treatments. The critical roles of ferroptosis in decreasing the activity of tumor cells and suppressing the growth of tumors have been demonstrated in recent studies. Ferroptosis, in combination with immune checkpoint inhibitors, shows synergistically enhanced antitumor efficacy [45]. Therefore, it is of great significance to further explore the mechanism underlying the immune-mediated induction of ferroptosis to improve the efficacy of immunotherapy for patients with ccRCC.

In the present study, a novel FRLs signature consisting of seven lncRNAs (AC006129.2, CTB-41I6.2, CTD-2510F5.4, RP5-994D16.9, RP11-298J20.4, CTD-2396E7.11, and TUG1) was established for ccRCC. Based on this signature, the high-risk group had a significantly worse overall survival than the low-risk group. Univariate and multivariate Cox regression analyses revealed the risk score as a significant risk factor. Moreover, the current study provides the first comprehensive analysis of the crucial roles of FRLs in the prognosis, immune cell infiltration, and m6A modifications in patients with ccRCC. We first elucidated the correlation between the FRLs signature and the immune landscape; the differential immune analysis results demonstrated that risk score was closely linked to the proportion of infiltrated immune cells in this study. Moreover, the analysis of immune checkpoint and m6A genes of patients with ccRCC in the two risk groups indicated that the risk score was significantly associated with immune checkpoint and m6A genes; the differential expression of these genes correlated with the survival of ccRCC patients in different risk groups. These results may provide a promising strategy with important clinical implications for guiding individual therapies.

As a recently discovered unique form of iron-dependent non-apoptotic cell death, ferroptosis is involved in regulating the occurrence and development of various diseases, including tumors [46, 47]. Therefore, targeting ferroptosis is a promising approach for inhibiting tumor growth and proliferation [48]. Xu et al. suggested that in addition to these key ferroptosis trigger signals, there is an intricate crosstalk between various tumor-related signaling pathways and ferroptosis [7]. In this study, we identified 54 ferroptosis-related DEGs. GO annotation and KEGG enrichment analysis revealed that these genes are primarily correlated with autophagy, the HIF-1 signaling pathway, and glutathione metabolism. Previous studies have demonstrated that autophagy plays an essential role in ferritin degradation and promoting ferroptosis by degrading ferritin in fibroblasts and tumor cells [49, 50]. Moreover, studies have reported that targeting the autophagy-mediated ARNTL-EGLN1-HIF1A pathway may enhance the anti-cancer activity of ferroptosis activators in human non-small cell lung cancer cell lines, namely Calu-1 and HT1080 (a human fibrosarcoma cell line) [51]. Stockwell et al. elucidated that glutathione peroxidase 4 detoxifies lipids using glutathione to inhibit ferroptosis [52].

As a form of iron-dependent programmed cell death, ferroptosis is regulated by various lncRNAs, miRNAs, and genes. Recent studies on the regulation of ferroptosis by lncRNAs have been conducted in various cancers, including ccRCC [53], bladder cancer [16], and hepatocellular carcinoma [54]. lncRNA prognostic signatures have been constructed and applied to forecasting the clinical outcomes of patients with various tumors, including ccRCC. However, the ferroptosis–lncRNA interaction in the ccRCC prognostic signature remains elusive. Here, for the first time, we constructed a prognostic signature based on seven FRLs (AC006129.2, CTB-41I6.2, CTD-2510F5.4, RP5-994D16.9, RP11-298J20.4, CTD-2396E7.11, and TUG1) to predict the prognosis, immune infiltration, and the expression of immune checkpoint and m6A genes in patients with ccRCC, providing a novel perspective for the prognosis and treatment of ccRCC.

Currently, an increasing number of signatures composed of FRLs have been used in the prognosis and diagnosis of various diseases, including sepsis, glioma, liver cancer, and bladder cancer [55–58]. Increasing evidence has confirmed that lncRNAs play an important role in the occurrence, diagnosis, and treatment of a variety of diseases [59–61]. The results of this study suggested that the seven FRLs that constitute our prognostic signature have important prognostic value in ccRCC. Furthermore, several previous independent reports have confirmed the biological function of candidate lncRNAs in ccRCC pathology. For instance, Lv et al. confirmed that TUG1 promoted ccRCC cell proliferation and inhibited apoptosis and autophagy by regulating the miR-31-5p/FLOT1 axis [62]. Previous studies have shown that TUG1 is significantly associated with histological grade, tumor stage, lymph node metastasis, and distant metastasis of ccRCC [63]. Moreover, another study has presented that CTD-2510F5.4 is a malignant phenotype-associated lncRNA that regulates cell cycles and apoptosis in gastric cancer [64]. Another literature revealed that high levels of CTD-2510F5.4 correlated with poor prognosis in patients with hepatocellular carcinoma [56]. Zheng et al. reported that CTD-2396E7.11 is an independent risk prognostic factor for ovarian cancer [65].

There is complex crosstalk between ferroptosis and tumor immunity. A previous study suggested that dying ferroptosis cells can release and activate damage-associated molecular patterns or lipid oxidation products in immune cells, such as macrophages, monocytes, and neutrophils, through different intracellular signaling pathways, resulting in different immune and inflammatory responses [66]. Another significant contribution of the present study is highlighting the correlation between our FRLs-based signature and tumor immune microenvironment. In this study, the ferroptosis-related differentially expressed genes were enriched in many immune-related BPs and pathways, including the T cell apoptotic process, IL-17 signaling pathway, and Th17 cell differentiation (Additional file 3: Table S3). Based on the results of our study, the FRLs signature is associated with immune infiltration in ccRCC, and these FRLs may be a target for combination therapy with immune checkpoint inhibitors. In addition, CIBERSORT analysis revealed higher proportions of M0 macrophages, Tregs, activated CD4 memory T cells, CD8 T cells, and follicular helper T cells infiltration in the high-risk group, and higher proportions of M2 macrophages, naive B cells, resting dendritic cells, resting monocytes, resting mast cells, and neutrophil infiltration in the low-risk group. These results suggest that the FRLs signature correlated with the immune landscape of the ccRCC microenvironment. Nevertheless, the mechanisms underlying the relationship between ferroptosis and ccRCC immunity warrant further investigation.

In the present study, a total of 14 immune checkpoint genes (*PDCD1* (*PD-1*), *CD274* (*PD-L1*), *CTLA4*, *ADORA2A*, *C10orf54* (*VISTA*), *HAVCR2* (*TIM-3*), *ICOSLG*, *NT5E*, *CD27*, *IDO2*, *LAG3*, *TIGIT*, *TNFRSF18*, and *TNFRSF9*) and nine m6A regulators (*METTL14*, *WTAP*, *FTO*, *ALKBH5*, *YTHDF1*, *YTHDF2*, *YTHDF3*, *YTHDC1*, and *YTHDC2*) were differentially expressed between the two groups of patients with ccRCC. Tang et al. have demonstrated that the induction of ferroptosis in combination with immune checkpoint inhibitors cooperatively enhances antitumor activity [44]. Consistently, Fan et al. developed an inhibitor, BEBT-908, which dual-targeted PI3K and HDAC, effectively inhibiting tumor cell growth and enhancing anti-PD1 therapy in mice by inducing immunogenic ferroptosis in cancer cells [67]. These results suggest that the synergistic effect of ferroptosis and immune regulation results in effective antitumor activity. Additionally, several studies have reported abnormal m6A modifications in the ferroptosis process in various diseases [68]. A previous study showed that decreased ALKBH5 mRNA levels correlated with shortened overall and cancer-specific survival in ccRCC [69]. Xu et al. suggested that METTL3-mediated m6A modification can stabilize SLC7A11 mRNA and facilitate its translation, thereby promoting cell proliferation and inhibiting cellular ferroptosis of lung adenocarcinoma [70]. These results provide a theoretical basis and indicate that m6A modification may play a promising role in ferroptosis and is correlated to cancer prognosis. Further studies are necessary to obtain more details on the potential associations.

However, our study had some limitations. First, our analysis incorporated a large number of ccRCC samples from TCGA and randomly divided the data into training and validation sets to improve the robustness of the study. The FRLs prognostic signature should be further verified in future studies. Second, our study proposes a significant correlation between the FRLs signature and immune infiltration in ccRCC; however, the potential regulatory mechanism requires further elucidation by functional experiments. Third, although the estimation bias of CIBERSORT is considerably lower than that of other methods, CIBERSORT analysis relies on limited genetic data, so its results may be affected by atypical interactions of cells, disease-induced disease, or phenotypic plasticity and tend to systematically overestimate or underestimate some cell types [71, 72]. Therefore, experiments will be carried out to further verify the prognosis model, the results of immune infiltration, and their regulatory mechanisms in our future studies.

## Conclusions

In conclusion, our study constructed a signature consisting of seven FRLs, which effectively predicted the prognosis and immune infiltration state of patients with ccRCC, and further analyzed the immune checkpoint and m6A gene expression in patients with different risk stratifications, thus providing a new direction for individualized, targeted therapy, which has many potential prognostic and therapeutic implications for the management of patients with ccRCC.

## Supplementary Information

**Additional file 1. Table S1.** The list of 259 ferroptosis-related genes.

**Additional file 2. Table S2.** The list of 65 lncRNA-gene pairs.

**Additional file 3. Table S3.** The results of GO and KEGG enrichment analysis of ferroptosis-related differentially expressed genes.

**Additional file 4: Fig. S1.** Survival analysis of seven FRLs constituting the prognostic signature in the training set. The KM curves indicate that there were significant survival differences between patients with high and low expression of CTD-2396E7.11 (A), RP11-298J20.4 (B), RP5-994D16.9 (C), CTD-2510F5.4 (D), CTB-41I6.2 (E), AC006129.2 (F), and TUG1 (G) in the training set.

**Additional file 5: Fig. S2.** Survival analysis of seven FRLs constituting the prognostic signature in the validation sets. The KM curves indicate that there were significant survival differences between patients with high and low expression of CTD-2396E7.11 (A), RP11-298J20.4 (B), RP5-994D16.9 (C), CTD-2510F5.4 (D), CTB-41I6.2 (E), AC006129.2 (F), and TUG1 (G) in the validation set.

**Additional file 6: Fig. S3.** DCA analysis of the training and validation sets. The 1 (A), 3 (B), and 5 (C) years decision curves of the training set indicate that the clinical utility of the prognosis model is superior to other clinical parameters. The 1 (D), 3 (E), and 5 (F) years decision curves of the validation set indicate that the clinical utility of the prognosis model is superior to other clinical parameters.

**Additional file 7: Fig. S4.** Analysis of immune infiltration in ccRCC patients in the validation set. (A) Boxplots showing infiltration of immune cells in ccRCC patients from the two groups in the validation set. (B) Bubble plots representing the correlations between immune cells and the risk score. Scatter plot of the correlation between the risk score and various immune cells, including regulatory T cells (Tregs) (C), monocytes (D), activated CD4 memory T cells (E), CD8 T cells (F), follicular helper T cells (G), and M2 macrophages (H). *p < 0.05, **p < 0.01, ***p < 0.001.

## Data Availability

Publicly available datasets were analysed in this study. This data can be downloaded here: UCSC Xena: GDC TCGA Kidney Clear Cell Carcinoma(KIRC)(https://xenabrowser.net/datapages/?dataset=TCGA-KIRC.htseq_fpkm.tsv&host=https%3A%2F%2Fgdc.xenahubs.net&removeHub=https%3A%2F%2Fxena.treehouse.gi.ucsc.edu%3A443).

## Abbreviations

ccRCC: Clear cell renal cell carcinoma
lncRNAs: Long non-coding RNAs
FRLs: Ferroptosis-related lncRNAs
TGCA: The Cancer Genome Atlas
RCC: Renal cell carcinoma
BP: Biological process
MF: Molecular function
CC: Cellular component
m6A: N6-methyladenosine
AUC: Area under the curve
ROC: Receiver operating characteristic
DCA: Decision curve analysis
LASSO: Least absolute shrinkage and selection operator
DEGs: Differentially expressed genes
DE-lncRNAs: Differentially expressed lncRNAs
GO: Gene Ontology
KEGG: Kyoto Encyclopedia of Genes and Genomes
