# Supplementary figures and images for: Construction of a ferroptosis-related signature based on seven lncRNAs for prognosis and immune landscape in clear cell renal cell carcinoma
Source: BMC Med Genomics. 2022 Dec 17;15:263. doi: 10.1186/s12920-022-01418-2 (PMC9758795; doi:10.1186/s12920-022-01418-2)

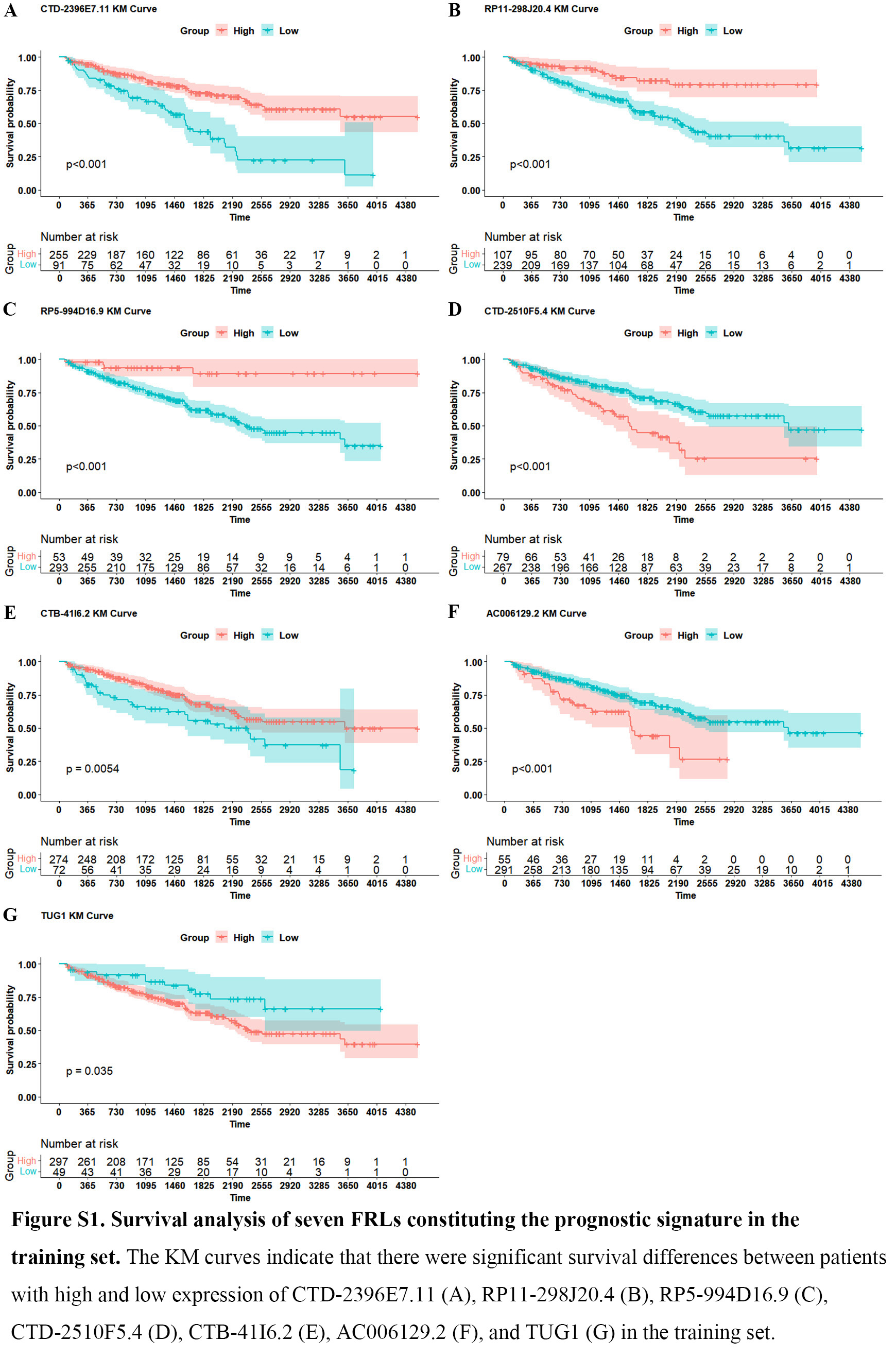

Supplement: Supplementary file 4 — Additional file 4: Fig. S1. Survival analysis of seven FRLs constituting the prognostic signature in the training set. The KM curves indicate that there were significant survival differences between patients with high and low expression of CTD-2396E7.11 (A), RP11-298J20.4 (B), RP5-994D16.9 (C), CTD-2510F5.4 (D), CTB-41I6.2 (E), AC006129.2 (F), and TUG1 (G) in the training set. [file 12920_2022_1418_MOESM4_ESM.jpg]

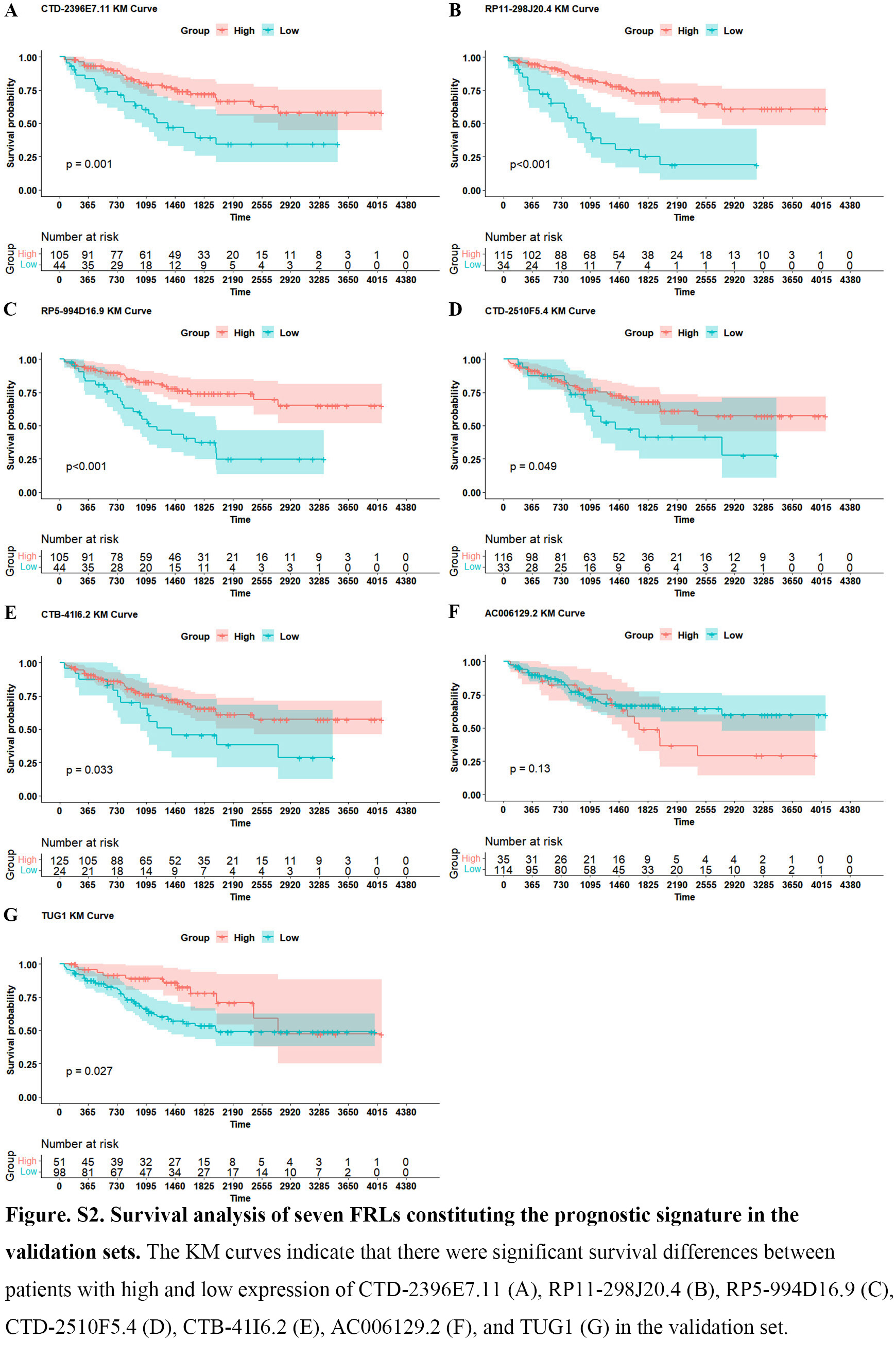

Supplement: Supplementary file 5 — Additional file 5: Fig. S2. Survival analysis of seven FRLs constituting the prognostic signature in the validation sets. The KM curves indicate that there were significant survival differences between patients with high and low expression of CTD-2396E7.11 (A), RP11-298J20.4 (B), RP5-994D16.9 (C), CTD-2510F5.4 (D), CTB-41I6.2 (E), AC006129.2 (F), and TUG1 (G) in the validation set. [file 12920_2022_1418_MOESM5_ESM.jpg]

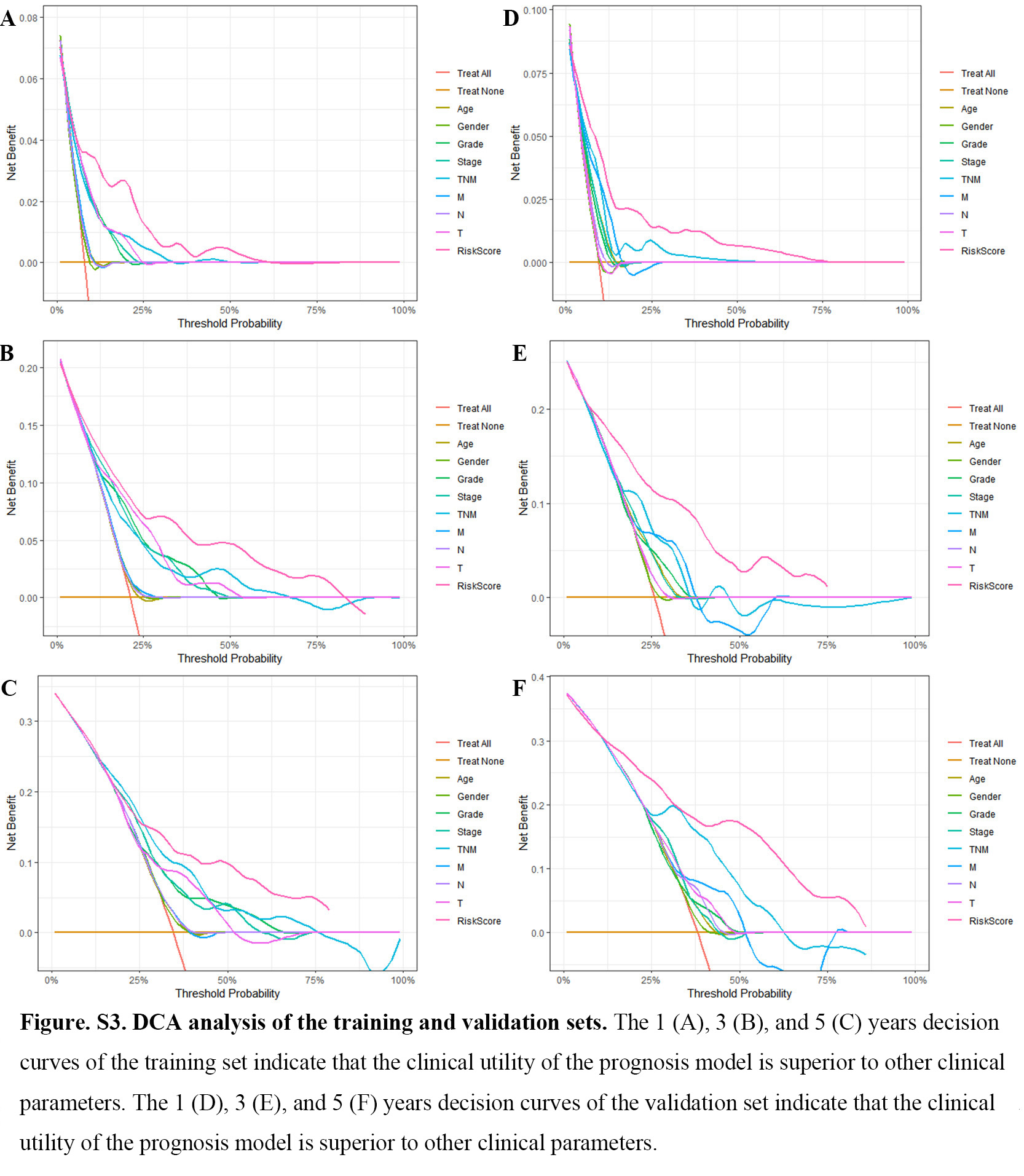

Supplement: Supplementary file 6 — Additional file 6: Fig. S3. DCA analysis of the training and validation sets. The 1 (A), 3 (B), and 5 (C) years decision curves of the training set indicate that the clinical utility of the prognosis model is superior to other clinical parameters. The 1 (D), 3 (E), and 5 (F) years decision curves of the validation set indicate that the clinical utility of the prognosis model is superior to other clinical parameters. [file 12920_2022_1418_MOESM6_ESM.jpg]

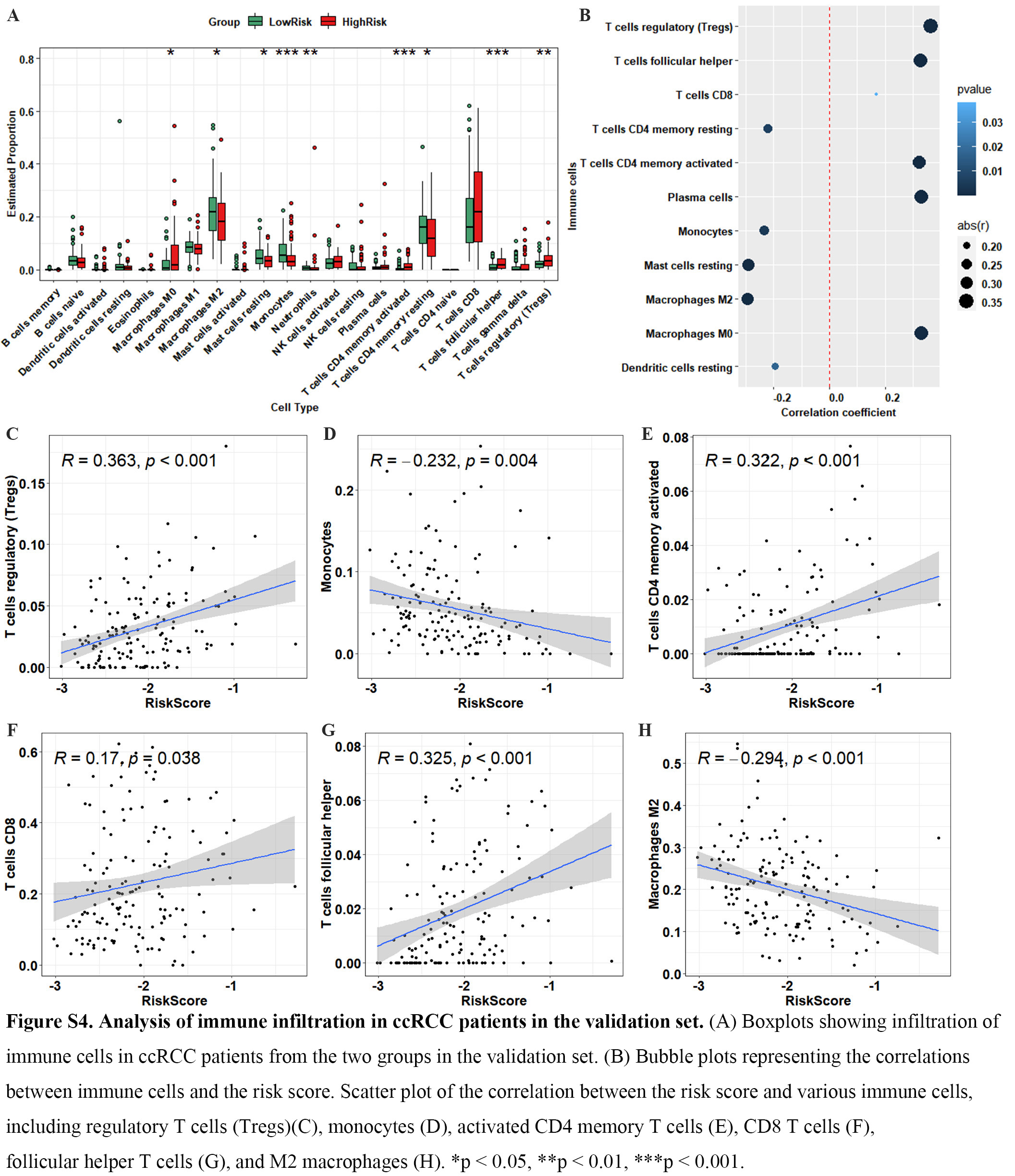

Supplement: Supplementary file 7 — Additional file 7: Fig. S4. Analysis of immune infiltration in ccRCC patients in the validation set. (A) Boxplots showing infiltration of immune cells in ccRCC patients from the two groups in the validation set. (B) Bubble plots representing the correlations between immune cells and the risk score. Scatter plot of the correlation between the risk score and various immune cells, including regulatory T cells (Tregs) (C), monocytes (D), activated CD4 memory T cells (E), CD8 T cells (F), follicular helper T cells (G), and M2 macrophages (H). *p < 0.05, **p < 0.01, ***p < 0.001. [file 12920_2022_1418_MOESM7_ESM.jpg]
